# Adaptive aspects of maximizing in times of COVID-19: coping efforts linking maximization to well-being

**DOI:** 10.3389/fpsyg.2023.1268528

**Published:** 2024-01-05

**Authors:** Young Joo Jun, Incheol Choi, Joo Hyun Kim

**Affiliations:** ^1^Center for Happiness Studies, Seoul National University, Seoul, Republic of Korea; ^2^Department of Psychology, Seoul National University, Seoul, Republic of Korea; ^3^Institute of Health Policy and Management, Medical Research Center, Seoul National University, Seoul, Republic of Korea

**Keywords:** maximizing, coping strategies, well-being, COVID-19, inconsistent mediation

## Abstract

**Introduction:**

Maximization, the tendency to make the best choices by thoroughly searching and comparing alternatives, has long been considered a negative correlate of well-being. However, recently, it was proposed that having a maximizing tendency can lead to better coping efforts in some stressful situations and thus could be more adaptive. The objective of the present research was to demonstrate positive features of maximization and identify the coping strategies that mediate the relationship between maximization and well-being during the COVID-19 pandemic.

**Methods:**

A sample of 3,493 participants responded to an online survey from January 20 to October 11 of 2020. The 13-item Maximization Scale assessed individuals’ maximizing tendency in terms of the maximization index and its three subdimensions (i.e., high standards, alternative search, and decision difficulty). The use of four coping strategies (i.e., preventive measures, cognitive appraisal, self-distraction, and social connection) during the COVID-19 pandemic was assessed. Hedonic and eudaimonic aspects of well-being were measured. We developed a mediation model and examined both whether there was an indirect link between maximization and well-being through the coping strategies and whether there was a direct link between maximization and well-being.

**Results:**

Path analysis revealed negative direct associations between maximization measures (i.e., an index and three subdimensions) and well-being. In addition, significant indirect paths were found with varying directions depending on maximization dimensions and coping strategy types. There were positive indirect associations between the maximization index and well-being via preventive measures, between high standards and well-being through preventive measures and cognitive appraisal, and between alternative search and well-being through self-distraction and social connection. Negative indirect associations were found between decision difficulty and well-being through cognitive appraisal, self-distraction, and social connection.

**Discussion:**

The current study confirmed the existence of inconsistent mediation effects between maximization and well-being via coping and highlighted coping efforts as one of the positive aspects of maximization. Discussion addressed the double-edged effect of maximization on well-being and its beneficial nature in times of distress. Future studies should examine other potential situations and moderators that can delineate maximization’s various characteristics with a longitudinal design and samples from diverse backgrounds.

## 1 Introduction

People differ in the ways they make choices. Maximization is one of the intrapersonal factors that explain individual differences in decision-making. It consists of three subdimensions (i.e., high standards, alternative search, and decision difficulty; Schwartz et al., 2002; Nenkov et al., 2008)*. High standards* involve holding high standards both for oneself and things across the board. *Alternative search* refers to exhaustively going through available options before making up one’s mind. Finally, *decision difficulty* indicates having a hard time deciding on things such as a gift for a friend or the best wording for a letter. Maximizers with higher levels of such maximizing tendencies are individuals who strive to make the best choice through an exhaustive search and comparison of alternatives (Schwartz et al., 2002; Cheek and Schwartz, 2016). They also tend to put great effort into the decision process by comparing more options (Schwartz et al., 2002; Chowdhury et al., 2009; Dar-Nimrod et al., 2009; Polman, 2010; Yang and Chiou, 2010) and spending more time (Schwartz et al., 2002; Chowdhury et al., 2009; Misuraca and Teuscher, 2013). On the other hand, satisficers seek to choose options that are “good enough” and stop searching once they identify choices that satisfy their standards (Schwartz et al., 2002; Cheek and Schwartz, 2016).

Past research has drawn a pessimistic picture of maximizers in that maximizers tend to set unattainable goals and have problematic decision-making styles (e.g., Schwartz et al., 2002; Parker et al., 2007; Purvis et al., 2011; Hughes and Scholer, 2017). Regarding the influence of having a maximizing tendency on psychological well-being, it has been considered dysfunctional for general well-being due to its negative associations with various well-being indicators (i.e., life satisfaction, depression, and happiness; Schwartz et al., 2002; Iyengar et al., 2006; Parker et al., 2007; Cheek and Schwartz, 2016; Kim and Miller, 2017; Newman et al., 2018). For instance, compared to satisficers, maximizers are known to be less happy (Schwartz et al., 2002; Purvis et al., 2011), less optimistic (Schwartz et al., 2002), and less open (Purvis et al., 2011). They also report lower life satisfaction (Schwartz et al., 2002; Chang et al., 2011; Newman et al., 2018), have reduced self-esteem (Schwartz et al., 2002), and experience more regret (Schwartz et al., 2002; Lai, 2011; Purvis et al., 2011; Ma and Roese, 2014; Kokkoris, 2019). Furthermore, being a maximizer is positively correlated with perfectionism (Schwartz et al., 2002; Chang et al., 2011; Dahling and Thompson, 2013), depression (Schwartz et al., 2002; Bruine de Bruin et al., 2016) and often engaging in counterfactual thinking (Schwartz et al., 2002; Leach and Patall, 2013) and social comparison (Schwartz et al., 2002; Weaver et al., 2015; Cheek and Schwartz, 2016; Luan and Li, 2019). Iyengar et al. (2006), for example, showed that even though graduating college seniors with maximizing inclinations got jobs with higher salaries than seniors with satisficing inclinations, maximizers’ satisfaction levels were lower than those for satisficers.

But do maximizing tendencies always play a negative role in one’s well-being? In recent years, some researchers have begun to explore novel and positive aspects of maximizing tendencies and their positive effects on well-being (e.g., Kokkoris, 2016; Misuraca et al., 2016; Zhu et al., 2017; Peng et al., 2018; Schei et al., 2020; Brannon, 2021; Ma et al., 2021; Li et al., 2023). They highlighted maximizers’ greater level of future-oriented thinking (Misuraca et al., 2016; Zhu et al., 2017) and intrinsic and achievement motivation (Lai, 2011; Peng et al., 2018), qualities which have been regarded as valuable predictors of adaptability (e.g., Griffin and Hesketh, 2005; Bell and Kozlowski, 2008; Stokes et al., 2010; Anagnostopoulos and Griva, 2012). For example, Ma et al. (2021) found that the past-positive and future-time perspectives served as mediators of the positive relationship between maximization and meaning in life. In addition, in a recent study by Peng et al. (2018), feelings of regret and achievement motivation were considered to be significant intervening variables in the relationship between maximization and subjective well-being. They found that maximizing fostered achievement motivation, which in turn was positively associated with SWB, while it also negatively influenced SWB through feelings of regret. Li et al. (2023) also demonstrated the long-term positive influence of the maximizing tendency on adaptability and well-being by showing that student maximizers acquired more eudaimonic well-being, and enhanced eudaimonic well-being, in turn, helped them adapt better to their new college life.

In addition to these positive aspects of maximization, researchers identified coping attempts as prominent features manifested by those predisposed to maximizing (Schwartz et al., 2002); maximizers may put greater effort into coping with physical and psychological distress. Coping refers to behavioral and psychological efforts that people consciously employ to master, minimize, or tolerate stressors (Lazarus and Folkman, 1984), and in general, the use of coping mechanisms is known to be helpful in maintaining one’s well-being (Shiota and Levenson, 2012; Gross, 2015; Polizzi et al., 2020; Kim et al., 2022). Although some evidence suggests that there is a positive association between maximization and well-being indicators (e.g., meaning in life; Ma et al., 2021) and the mediators in the associations (e.g., achievement motivation; Peng et al., 2018), the mechanisms accounting for the relationship between maximization and well-being (defined by hedonic and eudaimonic well-being), particularly the role of coping efforts, remain understudied.

The COVID-19 pandemic was a specific context that allowed us to examine potential positive aspects of maximizing in relation to coping and well-being. Thus, in the present study we sought to identify the relationships among maximization, coping, and well-being by establishing a mediation model in which maximization is related to well-being through coping strategies.

### 1.1 Coping as a positive aspect of maximization

In the general discussion of the potential positive role of a maximizing strategy in maintaining well-being, Schwartz et al. (2002) endorsed the view that responding to health threats with a maximizing strategy, characterized by exhaustive information search and active efforts to acquire the best possible treatment, can lead to better outcomes than a strategy that simply selects a treatment considered sufficient. Utilizing active coping practices under stress is one example well representing the general tendency of maximizers’ future-oriented thinking — “the present anticipation of future goals and consequences of current actions” (Zhu et al., 2017, p. 94). Maximizers tend to give greater consideration to the future consequences of their actions and attend more to future goals than satisficers (Misuraca et al., 2016; Zhu et al., 2017). Such future-oriented thinking has been linked to greater engagement in proactive and adaptive coping strategies (Aspinwall, 2005, 2011; Sohl and Moyer, 2009; Anagnostopoulos and Griva, 2012; Chua et al., 2015). Chua et al. (2015) found that adolescents who express high levels of future orientation are more inclined than others to employ adaptive coping strategies while avoiding maladaptive ones as they work towards achieving positive future outcomes. Furthermore, maximizers tend to possess stronger aspirations and a more unwavering dedication to achieving favorable goals and success than those with lower maximizing tendencies (Peng et al., 2018; Zhu et al., 2022). Therefore, despite lots of demands such as planning and preparation (Iyengar et al., 2006; Polman, 2010), maximizers work harder (e.g., Schwartz et al., 2002), investing more time (Nenkov et al., 2008) and resources (Misuraca et al., 2016) while resisting temptations (Besharat et al., 2014) and risky behaviors (Lai, 2010) that may reduce the likelihood of goal achievement. Indeed, maximizers exhibit greater levels of achievement motivation (Peng et al., 2018), which leads to a stronger internal drive and motivation system for overcoming external challenges and addressing difficulties (Te Wang and Eccles, 2013; Michou et al., 2014). All these perspectives highlight the possibility that maximizers may utilize various coping strategies in times of crisis in order to improve the situation and secure a good future. The coping efforts, in turn, can bring better well-being.

During the COVID-19 pandemic, certain types of coping were more likely to be used due to the contagious nature of COVID-19. Kim et al. (2022) developed the eight items that have been widely implemented during the pandemic based on the Brief COPE Scale (Carver, 1997) and the distress regulation literature supporting their effectiveness (Folkman and Lazarus, 1980; Carver et al., 1989). The internal and external validity of these eight items were confirmed (Kim et al., 2022) by conducting exploratory factor analysis (EFA) and examining associations between coping strategies and changes in well-being in a longitudinal analysis. Three constructs were revealed in the EFA using the eight items: cognitive appraisal, behavioral strategies, and preventive measures. Cognitive appraisal represents cognitive efforts to reduce the stress or negative emotions aroused by the COVID-19 pandemic. Behavioral strategies refer to participation in activities or social interaction to avoid distress, and two items were highly loaded on this factor; ‘self-distraction’ involves participating in activities to take one’s attention away from stressors, and ‘social connection’ indicates communicating with people, even remotely (e.g., texting, phone calls), to activate one’s social support system, which was especially important during times of social distancing. Finally, preventive measures refer to preventive behaviors people employ to protect themselves from COVID-19, and include mask-wearing, hand hygiene, and social distancing. These constructs were found to be positively correlated with each other. Accordingly, the present study focused on these coping strategies to investigate their roles in mediating the association between maximization and well-being. The two items for behavioral strategies were considered separately to capture the nature of the activities, resulting in the assessment of four distinct coping strategies (i.e., cognitive appraisal, self-distraction, social connection, and preventive measures; Brooks et al., 2020; Polizzi et al., 2020; Kim et al., 2022).

We expected that maximizers would be more likely to practice these coping strategies to deal with physical and psychological distress during the pandemic. In particular, considering their concerns about the long-term consequences of their current actions (Misuraca et al., 2016; Zhu et al., 2017) and higher internal motivation to attain favorable outcomes (Peng et al., 2018; Zhu et al., 2022), they would be more willing to stick to public health rules and recommendations such as mask-wearing, hand hygiene, and social distancing and to attempt various activities in times of long-lasting pandemic crisis.

Yet, there were no *a priori* hypotheses about the relationships between specific maximization dimensions (i.e., high standards, alternative search, decision difficulty) and types of coping strategies. In fact, previous researchers have discovered different correlations between respective subdimensions and diverse psychological traits (Nenkov et al., 2008; Rim et al., 2011; Richardson et al., 2014). For example, Nenkov et al. (2008) found that while all three subdimensions had positive correlations with regret, only alternative search had a significant negative correlation with people’s satisfaction with life. Similarly, decision difficulty was the only dimension negatively related to optimism. Finally, high standards did not relate negatively to people’s happiness, optimism, or life satisfaction but were positively linked to perfectionism and the need for cognition (Nenkov et al., 2008). Given the distinct nature of each dimension and empirical findings on different relationships between the three subdimensions and various psychological traits, we decided to consider each maximization dimension in addition to global maximization in examining the mediation model and expected that each dimension would relate to coping strategies in different ways.

### 1.2 Coping strategies and well-being

Regarding the relationships between each coping strategy and well-being, engagement in cognitive appraisal, self-distraction, and social connection have all been found to be positively associated with well-being. For instance, cognitive appraisal has been shown to be helpful in decreasing the level of fear or anxiety (Folkman and Lazarus, 1980; Folkman et al., 1986). Reinterpreting and paying attention to positive sides by discovering lessons and meaning out of negative events can assist people in managing restrictions caused by the outbreak (Tennen and Affleck, 2002; Shiota and Levenson, 2012). Indeed, various studies have demonstrated the essential role of cognitive appraisal in dealing with psychological distress (Lazarus and Folkman, 1984; Carver and Scheier, 2012; Gross, 2015).

Self-distraction has also been found to be effective in lowering psychological distress and overcoming boredom and helplessness incurred by social isolation (Klinger, 1975; Polizzi et al., 2020). Lastly, during times of social distancing, connections with others, even remotely, have been shown to be effective ways to reduce psychological distress (Thoits, 1995; Kim et al., 2008, 2022). Social support systems activated by social connection can buffer negative emotions evoked by social distancing (Fiorillo and Gorwood, 2020; Holmes et al., 2020). As such, greater use of coping strategies during epidemic outbreaks can help maintain well-being.

Preventive measures have been recognized as effective ways to prevent the spread of the virus (Brooks et al., 2020; Ferguson et al., 2020). The high mortality rate and contagious nature of COVID-19 can make people feel fearful and insecure when out in public (Ahorsu et al., 2020). Moreover, higher engagement in measures such as wearing a mask, washing one’s hands, and refraining from close contact with others can be beneficial for maintaining mental and physical health. Unlike other coping strategies, however, it has been proposed that preventive measures may be accompanied by a cost to well-being. Kim et al. (2022) found that those who strictly followed preventive measures experienced a significant decrease in well-being during the first phase of the pandemic. Indeed, many have experienced difficulties while engaging in the preventive measures (Moore et al., 2021) and have refused to comply with the recommendations (Waldrop and Gallman, 2020). This study provides additional evidence regarding the relationship between practicing preventive measures and well-being during the COVID-19 pandemic.

### 1.3 The present study

The purpose of the present study is to investigate whether maximizers used higher levels of coping strategies and whether these coping attempts enhanced well-being during the COVID-19 pandemic. We predicted that maximizers would thoroughly search for optimal ways to manage the current situation and thereby would be inclined to explore and engage more in specific coping strategies. Ultimately, their increased attempts to cope with stress would help them to maintain a higher level of well-being. In addition to the hypothesized positive indirect paths, we did not overlook the previous findings that maximization has been negatively linked to well-being indicators (e.g., Schwartz et al., 2002; Cheek and Schwartz, 2016; Kim and Miller, 2017; Newman et al., 2018). Thus, when testing the mediation model, we can confirm the existence of an inconsistent pattern of mediation in which the direct and the indirect paths are significant and have opposite directional signs.

Inconsistent mediation (MacKinnon et al., 2000; Shrout and Bolger, 2002) is one example of partial mediation where the direct and the indirect paths have opposite signs, and it is distinct from a general mediation model in which both indirect and direct paths have the same direction. Inconsistent mediation is also called “competitive mediation” (Zhao et al., 2010) because the direct and indirect paths are competing with each other. This analytic approach has been widely used by scholars who explored the nature of various psychological traits (Kühnel et al., 2009; Widmer et al., 2012; Ang and Malhotra, 2016; Cauberghe et al., 2021; Zhou et al., 2022). For instance, Zhou et al. (2022) examined whether nostalgia counteracts loneliness by increasing happiness. In testing an inconsistent mediation model from loneliness to happiness via nostalgia using three different national samples, they found that although lonely people tended to experience decreased happiness (i.e., a negative direct path), they felt more nostalgia than less lonely people, and greater nostalgia, in turn, was associated with greater happiness (i.e., a positive indirect path). In the same vein, the present study tests an inconsistent mediation model involving a negative direct link between maximization and well-being and a positive indirect link between maximization and well-being through coping strategy.

It is also important to reiterate that the inconsistent mediation model was tested with the three subdimensions of maximization separately in addition to their summed maximization scores to identify more nuanced influences of maximization on coping efforts and well-being.

## 2 Materials and methods

### 2.1 Participants

A sample of 3,493 Koreans aged 14–71 (87.7% Female; *M*_age_ = 27.24, *SD*_age_ = 9.94) provided information on the variables of interest via Kakao online surveys (Table 1). Kakao Corporation is one of the biggest SNS companies in South Korea, and 86% of the Korean population uses it. Since 2019, the Center for Happiness Studies at Seoul National University has been collecting data on Koreans’ daily well-being and various psychological measures in collaboration with Kakao Corporation (see Choi et al., 2021 for detailed information on the data). The dataset has been widely used by researchers whose interests are to clarify the psychological and environmental mechanisms underlying well-being (Choi et al., 2021; Na et al., 2021; Suk et al., 2021; Kim et al., 2022). Any Kakao user can voluntarily participate in the survey at any time through its website^1^ or application (i.e., KakaoTalk). Participants indicated their gender and birth year when they first signed up to participate in the survey. See Table 1 for information about participants’ characteristics.

**Table 1:** Demographic characteristics (*N* = 3,493).

	*N*	%
Gender		
Female	3,062	87.7
Male	431	12.3
Age		
10s	1,193	34.2
20s	1,392	39.9
30s	525	15.0
40s	271	7.8
50s	92	2.6
over 60s	20	0.6
Regions		
Capital	2,028	58.1
Others	1,465	41.9
Total	3,493	100

**Figure 1:**
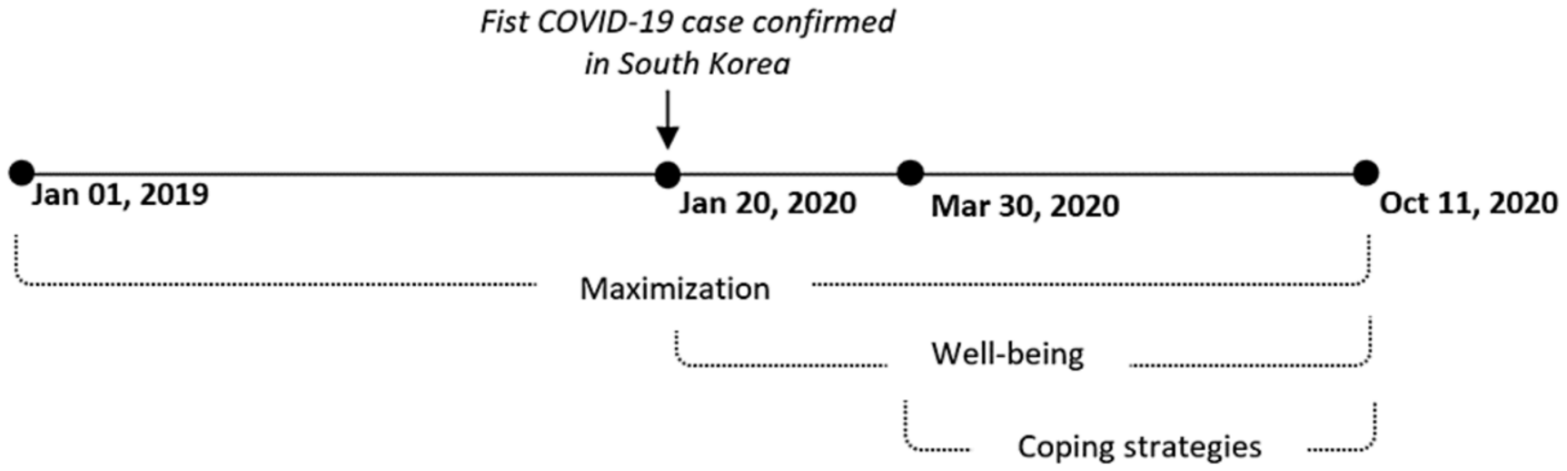
Study period and measures.

This study used the responses provided during the study period shown in Figure 1. Since users were allowed to respond to the survey items multiple times, well-being responses obtained more than once from each participant were averaged (The number of responses on well-being per person: *M* = 5.92, *SD* = 6.66). For maximization and coping measures, only the first response was used because most participants only responded once (The number of responses per person was: *M* = 1.21, *SD* = 0.79 for maximization; *M* = 1.08, *SD* = 0.39 for coping). The secondary data analysis of the Kakao data was approved by the Institutional Review Board at Kangwon National University in South Korea (#201910009002).

### 2.2 Measures

#### 2.2.1 Well-being

Well-being was measured using a 10-item well-being questionnaire, assessing hedonic well-being (i.e., life satisfaction, stress, positive affect, and negative affect) and eudaimonic well-being (i.e., meaning in life) (Diener, 1984; Diener et al., 1985; Ryff and Singer, 1998). Previous studies have validated the 10-item index among the Korean population and found excellent internal consistency for the selected items (Choi et al., 2021; Suk et al., 2021).

Life satisfaction, meaning in life, and stress were each assessed with a single question, respectively (e.g., “How satisfied are you with your life right now?” was the measure of life satisfaction). For positive and negative affect, participants were asked the extent to which they felt each of three positive (PA: happy, pleasant, relaxed) and four negative (NA: bored, annoyed, depressed, anxious) emotions, respectively. Participants responded on an 11-point Likert scale, ranging from 0 = not at all to 10 = very much (see Supplementary materials for the full survey items). The well-being index was created by averaging the ten items with reversed scores for stress and negative affect (*α* = 0.912).

#### 2.2.2 Maximization

The 13-item Maximization Scale (MS; Schwartz et al., 2002) is composed of three subdimensions (i.e., high standards, alternative search, and decision difficulty). The high standards subscale consisted of three items (e.g., “I never settle for second best.”), asking about the standards people have both for themselves and life events. Alternative search included six items (e.g., “Although I am satisfied with my current job, I try to look for better opportunities.”), asking whether people seek better options. Lastly, decision difficulty was measured with four items (e.g., “It is hard to choose which movie to watch.”), representing whether people find it hard to decide on things such as a gift for a friend or the best words to say. Participants responded on a 7-point scale (1 = completely disagree, 7 = completely agree). The present study used a composite score averaging across all 13 items (*α* = 0.677) and three subscale scores averaging items on each subscale (*α* = 0.580 for high standards, *α* = 0.574 for alternative search, and *α* = 0.585 for decision difficulty). The index and each MS dimension fall in the acceptable range of internal consistency (Nunnally, 1978; Kline, 2000).

#### 2.2.3 Coping strategies

Individuals’ coping strategies used during the pandemic (i.e., preventive measures, cognitive appraisal, self-distraction, and social connection) were measured with eight items developed based on the revised Brief COPE Scale (Carver, 1997). The Brief COPE contains measures of 12 coping strategies with each measure including two items. It has been widely used with a few modifications to reflect responses to specific stress-evoking situations such as the COVID-19 pandemic (e.g., Cauberghe et al., 2021). Accordingly, Kim et al. (2022) developed a scale consisting of eight items measuring coping strategies relevant to the COVID-19 pandemic, and they confirmed its internal and external validity (Kim et al., 2022).

Participants were asked to indicate the extent to which they used each coping strategy and responded on a 5-point scale, ranging from 1 = strongly disagree to 5 = strongly agree. In the current study, we averaged responses on three items for preventive measures (e.g., “I’ve been wearing a mask to prevent from the COVID-19.”) (*α* = 0.693) and three items for cognitive appraisal (e.g., “I’ve been trying to view the COVID-19 outbreak in a positive way.”) (*α* = 0.564), respectively. Social connection and self-distraction were measured with single items (“I’ve been communicating with my family and friends via phone/texts more than usual.” and “I’ve been doing other activities to take my mind off COVID-19.”, respectively). Thus, the use of four different coping strategies was measured.

### 2.3 Analytic strategy

We tested two models of the mediation relationships between maximization and well-being via coping strategies: one with the maximization index and the other with maximization’s three subdimensions. Path analysis was performed in Mplus Version 7.3. In the path analysis, one type of structural equation modeling (SEM) in which the model variables are observed, all variables of interest were entered into the model. Then, the indirect paths amongst the model variables and the direct relationships between variables were generated simultaneously.

The model variables satisfied the normality assumption (i.e., skewness <3; kurtosis <10) (Kline, 2011) (see Table 1), and maximum likelihood (ML) estimation was used to estimate the model parameters. Goodness-of-fit indices used to evaluate the path model included chi-square (nonsignificant), CFI (comparative fit index ≥0.95), RMSEA (root mean square error of approximation ≤0.06), and SRMR (standardized root mean square residual ≤0.08) (Hu and Bentler, 1999). The significance of the indirect paths was confirmed using 10,000 bootstrapped samples. According to previous findings that men are known to report higher levels of maximization compared to women (Iyengar et al., 2006; Parker et al., 2007), and older adults tend to engage less in maximizing decision strategies (Tanius et al., 2009; Bruine de Bruin et al., 2016), gender and age were statistically adjusted in the mediation analyses. The cutoff for statistical significance was set at α = 0.05. Table 2 presents the descriptive statistics for all study variables, and Table 3 presents the correlations among the study variables.

**Table 2:** Descriptive statistics (*N* = 3,493).

Variables	Mean	SD	Median	Range	Min	Max	Skewness	Kurtosis
*Maximization*								
Maximization index	4.22	0.94	4.23	6.00	1.00	7.00	0.10	0.17
High standards	4.73	1.21	4.67	6.00	1.00	7.00	−0.27	−0.04
Alternative search	4.05	1.05	4.00	6.00	1.00	7.00	0.13	0.09
Decision difficulty	4.10	1.32	4.00	6.00	1.00	7.00	0.03	−0.36
*Coping strategies*								
Preventive measures	4.17	0.77	4.33	4.00	1.00	5.00	−1.08	1.48
Cognitive appraisal	3.45	0.87	3.33	4.00	1.00	5.00	−0.04	−0.43
Self-distraction	3.42	1.27	4.00	4.00	1.00	5.00	−0.43	−0.86
Social connection	2.94	1.36	3.00	4.00	1.00	5.00	0.09	−1.18
*Well-being*	5.24	2.00	5.10	10.00	0.00	10.00	−0.02	0.03

**Table 3:** Correlations among main study variables (*N* = 3,493).

Variable	1	2	3	4	5	6	7	8
*Well-being*								
1. Well-being index	–							
*Maximization*								
2. Maximization index	−0.21***	–						
3. High standards	−0.13***	0.71***	–					
4. Alternative search	−0.17***	0.85***	0.43***	–				
5. Decision difficulty	−0.21***	0.81***	0.46***	0.48***	–			
Coping								
6. Preventive measures	0.10***	0.06***	0.12***	0.02	0.04*	–		
7. Cognitive appraisal	0.24***	−0.01	0.03	0.00	−0.04*	0.15***	–	
8. Self-distraction	0.26***	0.04*	0.02	0.07***	−0.01	0.14***	0.24***	–
9. Social connection	0.23***	0.03	0.02	0.07***	−0.03*	0.13***	0.13***	0.29***

Note. *p < 0.05, ***p < 0.001.

## 3 Results

### 3.1 Mediation paths from a maximization index to well-being via coping

**Figure 2:**
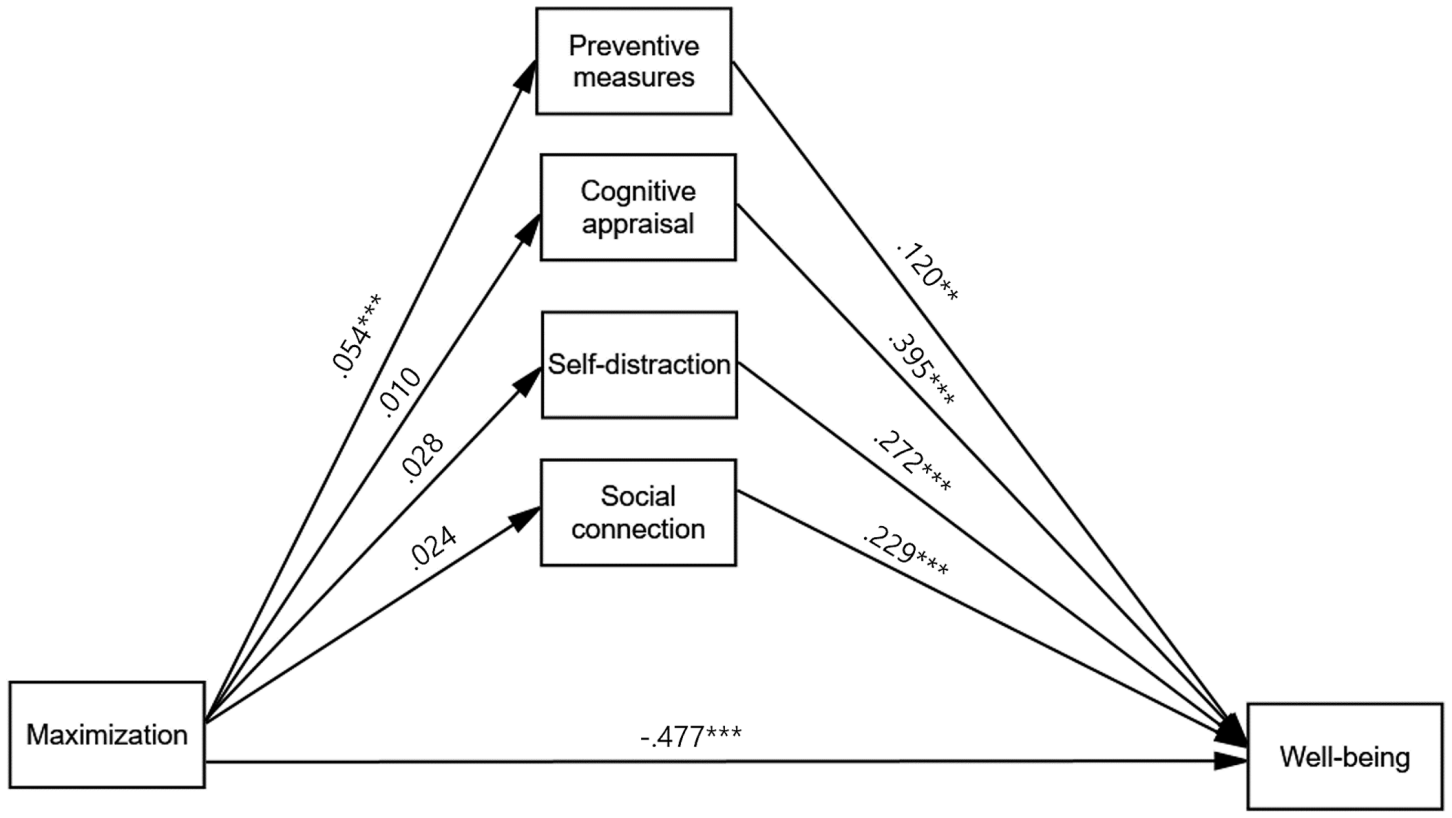
Parallel mediation model from a maximization index to well-being via coping strategies. Unstandardized estimates are present. Model fit: *χ*^2^ [3] = 6.464, *p* = 0.091, CFI = 0.998, TLI = 0.977, RMSEA = 0.018, SRMR = 0.005. Statistical controls include sex (Male = 1) and two age groups (Reference group: Age_10s–20s,_ Comparison groups: Age_30s–40s_, Age_50s and over_), but they were not depicted in the figure for model simplicity. * *p* < 0.05, ** *p* < 0.01, ***p < 0.001.

We examined whether the relationship between maximization and well-being is mediated by coping strategies. The path model linking the overall maximization index to well-being via four coping strategies showed a good fit to the data according to model indices (*χ*^2^[3] = 6.464, *p* = 0.091, CFI = 0.998, TLI = 0.977, RMSEA = 0.018, SRMR = 0.005) (see Figure 2). Results revealed a negative direct effect of maximization on well-being (*b* = −0.477, *SE* = 0.036, *p* < 0.001), indicating that those with higher maximization tendencies tend to have lower levels of well-being. However, more importantly, we found that preventive measures significantly and positively mediated the maximization and well-being link; those who scored higher on maximization followed the preventive measures more actively (*b* = 0.054, *SE* = 0.015, *p* < 0.001), which in turn had a positive effect on well-being (*b* = 0.120, *SE* = 0.043, *p* = 0.006) (Indirect effect: *b* = 0.007, *SE* = 0.003, *p* = 0.030, 95% CI = [0.001, 0.012]) (Figure 2; Table 4). The significance of the indirect relation was confirmed with 10,000 bootstrapped samples (Table 4). No additional significant mediation relationships were found for the other three coping strategies (i.e., cognitive appraisal, self-distraction, and social connection). Overall, we confirmed the inconsistent mediation paths, including both a negative direct path and a positive indirect path.

**Table 4:** Bootstrapped estimates of unstandardized total, total indirect, and indirect effects from three subdimensions of maximization (or maximization index) to well-being via coping strategies.

	Total effect (IV-DV)			Total indirect effect (IV-M and other Ms-DV)			Indirect effect (IV-M-DV)		
	*b* (*SE*)	95% CI	*p*	*b* (*SE*)	95% CI	*p*	*b* (*SE*)	95% CI	*p*
IV: Maximization M: Preventive measures DV: Well-being	−0.454*** (0.040)	−0.532, −0.375	0.000	0.023 (0.015)	−0.006, 0.053	0.124	0.007* (0.003)	0.001, 0.012	0.030
IV: High standards M: Preventive measures DV: Well-being	−0.044 (0.034)	−0.111, 0.023	0.200	0.033* (0.013)	0.007, 0.059	0.013	0.011* (0.004)	0.002, 0.020	0.013
IV: High standards M: Cognitive appraisal DV: Well-being	−0.044 (0.034)	−0.111, 0.023	0.200	0.033* (0.013)	0.007, 0.059	0.013	0.013* (0.006)	0.001, 0.026	0.031
IV: Alternative search M: Self-distraction DV: Well-being	−0.151*** (0.043)	−0.235, −0.067	0.000	0.059*** (0.016)	0.029, 0.090	0.000	0.029*** (0.007)	0.014, 0.043	0.000
IV: Alternative search M: Social connection DV: Well-being	−0.151*** (0.043)	−0.235, −0.067	0.000	0.059*** (0.016)	0.029, 0.090	0.000	0.027*** (0.007)	0.013, 0.041	0.000
IV: Decision difficulty M: Cognitive appraisal DV: Well-being	−0.239*** (0.035)	−0.307, −0.171	0.000	−0.056*** (0.012)	−0.080, −0.033	0.000	−0.013* (0.006)	−0.025, −0.001	0.027
IV: Decision difficulty M: Self-distraction DV: Well-being	−0.239*** (0.035)	−0.306, −0.172	0.000	−0.056*** (0.012)	−0.080, −0.033	0.000	−0.020** (0.006)	−0.032, −0.008	0.001
IV: Decision difficulty M: Social connection DV: Well-being	−0.239*** (0.035)	−0.306, −0.172	0.000	−0.056*** (0.012)	−0.080, −0.033	0.000	−0.023*** (0.006)	−0.034, −0.012	0.000

Note. *p < 0.05, **p < 0.01, ***p < 0.001.

### 3.2 Mediation paths from maximization subdimensions to well-being via coping

**Figure 3:**
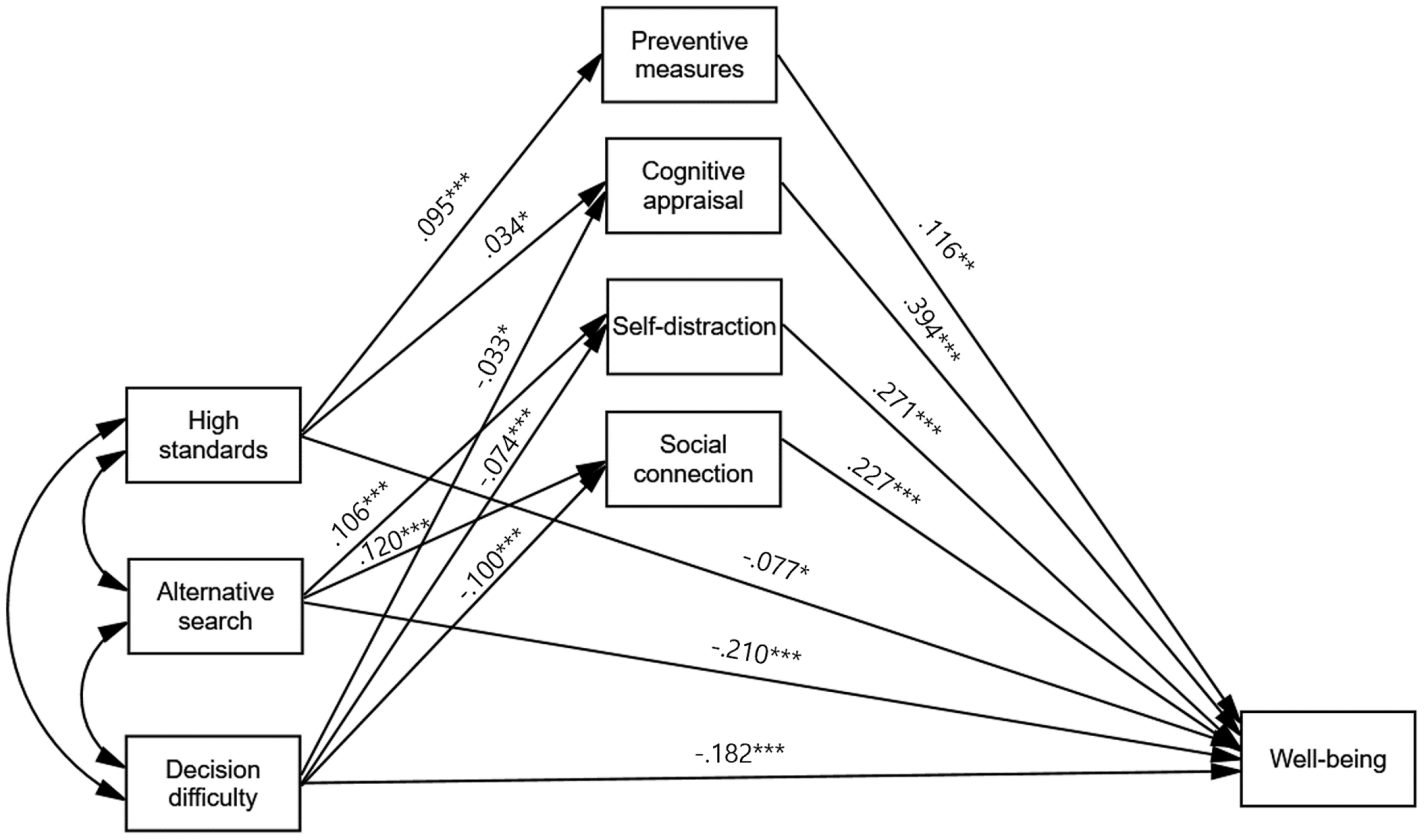
Parallel mediation model from three subdimensions of maximization to well-being via coping strategies. Only significant unstandardized estimates are present. Model fit: *χ*^2^ [5] = 17.733, *p* = 0.003, CFI = 0.992, TLI = 0.937, RMSEA = 0.027, SRMR = 0.007. Statistical controls include sex (Male = 1) and two age groups (Reference group: Age_10s–20s,_ Comparison groups: Age_30s–40s_, Age_50s and over_), but they were not depicted in the figure for model simplicity. *p < 0.05, **p < 0.01, ***p < 0.001.

Next, we examined the path model with three subdimensions of maximization. The model also showed a good fit to the data (*χ*^2^ [5] = 17.733, *p* = 0.003, CFI = 0.992, TLI = 0.937, RMSEA = 0.027, SRMR = 0.007) (Figure 3).

Consistent with the path model with a maximization index, there was a negative direct relation between each maximization subdimension and well-being. Among the three maximization subdimensions, alternative search showed the strongest negative relationship to well-being (*b* = −0.210, *SE* = 0.038, *p* < 0.001), followed by decision difficulty (*b* = −0.182, *SE* = 0.032, *p* < 0.001) and high standards (*b* = −0.077, *SE* = 0.031, *p* = 0.014).

Further, we found that the association between maximization and well-being was significantly mediated by coping strategies. Notably, the significance and directions of the indirect paths depended on maximization subdimensions and coping strategy types (Figure 3; Table 4). For high standards, there were positive indirect associations between high standards and well-being through preventive measures and cognitive appraisal. Participants with higher standards practiced preventive measures more actively (*b* = 0.095, *SE* = 0.013, *p* < 0.001) and used more cognitive appraisal strategies (*b* = 0.034, *SE* = 0.015, *p* = 0.027), which, in turn, led to higher levels of well-being (*b* = 0.116, *SE* = 0.044, *p* = 0.008 from preventive measures to well-being, *b* = 0.394, *SE* = 0.040, *p* < 0.001 from cognitive appraisal to well-being) (see Table 4 for bootstrapped estimates of indirect effects).

The relationship between alternative search and well-being was also positively mediated by self-distraction and social connection. Those scoring high on the alternative search subscale were more likely to use self-distraction (*b* = 0.106, *SE* = 0.025, *p* < 0.001) and social connection strategies (*b* = 0.120, *SE* = 0.028, *p* < 0.001), and greater use of those strategies was associated with increased well-being (*b* = 0.271, *SE* = 0.028, *p* < 0.001 from self-distraction to well-being, *b* = 0.227, *SE* = 0.026, *p* < 0.001 from social connection to well-being).

Decision difficulty was the only dimension of the maximization scale that showed negative indirect relationships to well-being through coping strategies. Decision difficulty negatively related to cognitive appraisal (*b* = −0.033, *SE* = 0.014, *p* = 0.021), self-distraction (*b* = −0.074, *SE* = 0.021, *p* < 0.001), and social connection (*b* = −0.100, *SE* = 0.022, *p* < 0.001). In addition, less use of these coping strategies during the pandemic reduced well-being.

Consistent with the previous model using the global maximization scale, we found inconsistent mediations linking three maximization subdimensions to well-being through coping strategies. Although those who scored high on high standards, alternative search, and decision difficulty tended to show lower well-being, the maximizing tendencies represented by high standards and alternative search led people to practice coping strategies more, and the increased coping efforts in turn enhanced well-being.

## 4 Discussion

This study is one of the first studies examining coping strategies as mediating mechanisms underlying the relationship between maximizing and well-being. We found that maximization, as measured by an overall maximization index and three subdimensions (i.e., high standards, alternative search, and decision difficulty), was negatively related to well-being. However, at the same time, higher maximization, excluding decision difficulty, led to greater use of coping strategies, which, in turn, increased well-being during the COVID-19 pandemic. The findings confirmed the inconsistent mediation between maximization and well-being via coping strategies, and showed that maximization has double-edged effects on well-being.

### 4.1 Positive aspects of maximizing tendency in the COVID-19 pandemic

Maximization has long had a negative reputation, with many studies showing negative relationships between maximization and well-being (e.g., Schwartz et al., 2002; Purvis et al., 2011; Oishi et al., 2014; Cheek and Schwartz, 2016). It is only recently that some scholars have raised skepticism regarding the negative associations and turned their attention to potential positive aspects of maximization (e.g., Kokkoris, 2016; Misuraca et al., 2016; Zhu et al., 2017; Peng et al., 2018; Schei et al., 2020; Brannon, 2021; Ma et al., 2021; Li et al., 2023). Li et al. (2023) found that students with a strong maximizing tendency have better adjustment skills after their first year of studies and attain higher GPAs upon completing their bachelor’s degrees. Peng et al. (2018) also demonstrated the inconsistent mediation between maximizing and subjective well-being (SWB). In their study, two competing indirect pathways simultaneously existed between maximizing and SWB. Specifically, maximizing often results in feelings of regret, which can have a negative impact on SWB. On the other hand, maximizing can also generate strong feelings of hope for success, which are positively associated with SWB.

The present study extended the findings of previous studies by examining possible positive indirect paths from maximization to well-being through coping strategies. Maximizers were likely to engage more in specific coping strategies to address the challenging situations caused by the COVID-19 pandemic. The increased use of coping strategies, in turn, positively affected their well-being. These findings are well aligned with the growing body of literature on the positive side of maximization tendencies. Maximizers have increased concern for the future impacts of their current actions (Misuraca et al., 2016; Zhu et al., 2017) and they desire to make the best choice among alternatives (Schwartz et al., 2002; Cheek and Schwartz, 2016). Schwartz et al. (2002) addressed that maximizers tend to show increased coping efforts under stressful situations and their maximizing strategies can give rise to positive outcomes. Their high achievement motivation (Peng et al., 2018), additionally, functions as a stronger internal drive for overcoming the difficulties they face (Te Wang and Eccles, 2013; Michou et al., 2014).

Our findings made an important contribution to the maximization literature by showing that maximizers have unique adaptive strengths, specifically greater motivation to deploy coping strategies, that shine during difficult times such as the pandemic. Moreover, our research demonstrated that the COVID-19 pandemic is a context in which the noble and positive aspects of maximization are highlighted. Previous studies have shown that a maximizing tendency can play a positive role, especially when a limited number of options is available. For instance, although a maximizing strategy undermines well-being as the size of the choice set becomes large, when there are only a small number of choices, a maximizing strategy may actually be the optimal choice strategy (Dar-Nimrod et al., 2009; Álvarez et al., 2014; Cheek and Schwartz, 2016). The COVID-19 pandemic was a situation in which only a restricted number of options were available to cope with the stressors. A maximizing strategy could be particularly helpful in such conditions. In addition, in the face of the COVID-19 pandemic, governments implemented strict regulations and urged citizens to adhere to a set of rules in order to prevent the spread of the disease and protect their citizens. According to trait activation theory, the relation between traits and performance differs based on the context, and one of the most straightforward ways to activate traits is by establishing expectations for desired behaviors within a group (Tett and Burnett, 2003). From this perspective, the pandemic situation, marked by high stress, uncertainty, and clear expectations for following regulations, was the optimal context for the activation of maximizing tendencies, especially in terms of coping. Our findings demonstrating maximizers’ greater coping efforts and their positive links to well-being during the pandemic support the idea that maximizing can, in part, be beneficial.

### 4.2 Differences in the mediation paths by maximization dimensions

The positive mediational links between maximization and well-being via coping strategies were only supported by the maximization index and two subdimensions (i.e., high standards and alternative search). For the decision difficulty subdimension, however, the mediation paths were negative, indicating that decision difficulty decreased the use of coping strategies and well-being. Additionally, each maximization dimension had significant relationships with different types of coping strategies. The maximization index only had a positive relationship with preventive measures, and use of preventive measures in turn was associated with greater well-being. High standards were positively associated with preventive measures and cognitive appraisal, while alternative search had positive associations with social connection and self-distraction. On the other hand, decision difficulty had negative relationships with cognitive appraisal, social connection, and self-distraction.

As shown in the present study, in times of high stress and uncertainty, such as the COVID-19 pandemic, a global maximizing tendency may lead people to set a prominent goal of safety from infection, and more willingly follow preventive measures to achieve the goal they set. This was the case for those with a higher score on high standards, one of the maximization subdimensions. The voluntary practice of preventive measures driven by internal maximizing motivation, not by external forces induced by the government, may not only reduce fear of infection but also satisfy individuals’ needs for safety and enhance their well-being. Previous research has indicated that practicing preventive measures can decrease well-being over time (Kim et al., 2022). However, the present study demonstrated that practicing preventive measures can enhance well-being. The different findings may be because of different study designs (e.g., longitudinal vs. cross-sectional) or target study periods during the pandemic. Future research should investigate whether and how practicing preventive measures affects our mental health by observing their use over a prolonged period of the pandemic and considering various underlying motivations. Another important finding of our study is that individuals who set and maintain high standards tended to use positive appraisal of adverse situations as one of the effective coping strategies to deal with difficulties, and this, in turn, increased well-being. Thus, maximizers’ strong motivation to achieve their goals and to deal with physiological and psychological distress is not limited to behavioral approaches but also includes cognitive efforts.

The nature of alternative search may drive people to exhaustively search for as many ways to cope with the negative consequences of COVID-19 as possible, such as engaging in activities or connecting with others remotely. These behavioral strategies were what people tried to alleviate heightened loneliness or boredom due to social distancing, and they were found to buffer the decrease in well-being over the initial period of the pandemic (Kim et al., 2022).

On the other hand, a tendency toward having difficulty with decisions may prevent people from actively dealing with the pandemic by leading them to just consider a variety of coping actions without ever deciding to adopt one and thereby losing chances to successfully mitigate stress caused by the pandemic. In fact, there has been a discussion on whether decision difficulty is a component of maximization or an outcome of it (e.g., Schwartz et al., 2002; Nenkov et al., 2008; Lai, 2010; Cheek and Schwartz, 2016; Cheek and Goebel, 2020). This study provided important empirical evidence on the construct of maximization; decision difficulty was linked to poor stress management skills, while high standards and alternative search were associated with greater coping efforts, ultimately leading to better mental health. This is in line with empirical evidence showing that maximizing has positive correlations with desired traits such as optimism, need for cognition, self-efficacy, and intrinsic motivation when the scale does not include a measure of decision difficulty (Lai, 2010).

The distinct mediation paths depending on subdimensions of maximization and different sets of relationships between maximization dimensions and types of coping strategies call for a fine-grained analysis that can delineate the distinctive nature of maximization dimensions.

### 4.3 Implications and strengths

Our findings demonstrate the need to explore the positive and unique features of various traits that may not ordinarily be thought of as positive. The maximizing tendency has long been viewed as maladaptive. Indeed, the vast majority of previous research has demonstrated negative associations between maximization and various well-being indicators (Schwartz et al., 2002; Iyengar et al., 2006; Parker et al., 2007; Chang et al., 2011; Dahling and Thompson, 2013; Oishi et al., 2014; Cheek and Schwartz, 2016). However, we identified adaptive aspects of maximizing that are helpful in times of crisis. Cheek and Schwartz (2016) demonstrated that novel aspects of having a maximizing tendency can be found when considering how maximizing goals and strategies interact with contextual factors and individual differences. Thus, future research should further examine potential situations and moderators that can explore maximization’s various characteristics. Similarly, it will be important for future research to explore various psychological traits in diverse contexts to uncover their unexpected features (e.g., loneliness; Zhou et al., 2022).

It is also important to note that our research investigated non-WEIRD (Western, educated, industrialized, rich, democratic) samples that have not been studied much in the maximization literature. In most previous maximization research, participants consisted of WEIRD samples (e.g., Schwartz et al., 2002; Diab et al., 2008; Richardson et al., 2014; Hughes and Scholer, 2017; Cheek and Ward, 2019). However, research studies limited to WEIRD cultures do not show a holistic picture of human psychology; instead, they may provide a biased understanding (Henrich et al., 2010). In addition, some preliminary studies have indicated that the relationships between maximization and well-being vary across cultures (Roets et al., 2012; Oishi et al., 2014; Datu, 2016). For instance, Roets et al. (2012) revealed that the maximizing tendency is a significant determinant of well-being in Western societies, but it still lacks a substantial association with well-being in non-Western societies. Thus, our attempt to clarify the role of maximization in maintaining well-being during the pandemic using a sample from South Korea provided another important piece of empirical evidence for understanding the mechanisms underlying the association between maximization and well-being.

### 4.4 Limitations and future directions

Our research, however, is not without limitations. First of all, the majority of participants in our sample were female (3,062 females, 87.7%) and young (2,585 people between 14 and 29 years of age, 74.0%). The survey was easily accessed through the Kakao application or website (see footnote 1), and any user could respond to the survey items at any time and multiple times. This way of data collection allowed us to collect a real-time dataset rather than retrospective responses on survey items. However, at the same time, due to voluntary participation, males and very old individuals were underrepresented in our sample. Although the absolute numbers of males (431 males, 12.3%), middle-aged individuals aged 30–49 years (796 people, 22.8%), and older adults aged 50–71 years (112 people, 3.2%) were not small, future research is needed that examines the relationships using more representative samples to increase generalizability.

Second, we only focused on the cross-sectional relationships among maximization, coping, and well-being. Although the maximizing tendency has been regarded as a dispositional decision style that remains relatively stable over time (Schwartz et al., 2002; Dar-Nimrod et al., 2009; Dalal et al., 2015), the design of our study did not permit the identification of cause-and-effect relationships among study variables. Employing a longitudinal approach can take into account the role of maximization in the dynamics of coping and well-being changes and clarify the causal relationships.

Finally, the current findings on the distinct nature of maximization dimensions in relation to coping efforts, especially decision difficulty—the only construct relating to poorer coping efforts—contained some surprises. Further research is warranted to more extensively examine the components of maximization. Indeed, there is still ongoing uncertainty regarding whether maximizing should be understood as a multi-dimensional or a unidimensional concept (Peng et al., 2018), and the measurement of maximizing has persistently presented a significant challenge for researchers within this domain (Cheek and Schwartz, 2016). While many different measures of maximizing have been created, there is no universally accepted approach to assessing it (Peng et al., 2018). Numerous researchers have discussed whether decision difficulty should be considered a component of maximization or a resultant outcome (e.g., Lai, 2010; Cheek and Schwartz, 2016; Cheek and Goebel, 2020). In forthcoming studies, alternative maximizing scales may be employed, specifically separating maximizing from decision difficulty, to clarify the relationship between maximizing and various psychological indicators.

## 5 Conclusion

During the pandemic, maximization served as a double-edged sword. On the one hand, maximization decreased well-being. However, on the other hand, having a maximizing tendency as assessed by the global index and two subdimensions (i.e., high standards and alternative search) led people to follow preventive measures more closely, appraise the situation positively, and find alternative ways to combat the negative consequences caused by the long-lasting pandemic situation by participating in various activities and making social connections. Ultimately, exploring and using these coping strategies, in turn, helped them to maintain greater well-being. The present study contributes to the growing body of research that addresses potential positive features of maximizing, and the findings on distinct mediation paths depending on subdimensions of maximization call for further studies on the constructs of maximization. This study also has public health implications regarding the importance of presenting a maximizing message during stressful situations such as the current pandemic. Based on our findings, future policymakers can utilize messaging to encourage maximization, highlighting the importance of aiming for the best, exploring various coping options, and putting into practice coping measures without hesitation to achieve greater well-being in times of crisis. An additional intriguing and underexplored area for potential future research is the identification of various contexts other than a pandemic in which a maximizing tendency predicts positive behaviors and outcomes. The potential situations and moderators that can delineate maximization’s various characteristics can be further investigated with different research designs (e.g., longitudinal design) and samples from diverse backgrounds. This approach will provide a more comprehensive perspective on maximization.

## Data availability statement

The data analyzed in this study is subject to the following licenses/restrictions: The dataset is not publicly available due to a non-disclosure agreement with the Kakao Corporation. However, the analysis dataset used for the present study is available from the Kakao Corporation (together@kakaocorp.com). Requests to access these datasets should be directed to together@kakaocorp.com.

## Ethics statement

The studies involving humans were approved by the Institutional Review Board (IRB) at Kangwon National University in South Korea. The studies were conducted in accordance with the local legislation and institutional requirements. The participants provided their written informed consent to participate in this study.

## Author contributions

YJJ: Conceptualization, Data curation, Formal analysis, Methodology, Writing – original draft, Writing – review & editing. IC: Formal analysis, Writing – review & editing. JHK: Data curation, Formal analysis, Methodology, Visualization, Writing – original draft, Writing – review & editing.

## Footnotes

^1^http://together.kakao.com/hello
