# Supplementary material for: Adaptive aspects of maximizing in times of COVID-19: coping efforts linking maximization to well-being
Source: Front Psychol. 2024 Jan 5;14:1268528. doi: 10.3389/fpsyg.2023.1268528 (PMC10797124; doi:10.3389/fpsyg.2023.1268528)
Supplement: Supplementary file 1 [file Table_1.docx]

# **Supplementary Materials**

# **Questionnaire Items***

## **Well-being**

To what extent do you agree with each of the following statements?

(0 = *not at all*; 10 = *very much)*

1. How satisfied are you with your life right now?
2. How happy are you right now?
3. How much meaning in life do you feel right now?
4. How stressed are you right now?
5. To what degree do you feel the following emotion? “bored”
6. To what degree do you feel the following emotion? “annoyed/frustrated”
7. To what degree do you feel the following emotion? “pleasant”
8. To what degree do you feel the following emotion? “relaxed”
9. To what degree do you feel the following emotion? “depressed”
10. To what degree do you feel the following emotion? “worried/anxious”

## **Maximization**

To what extent do you agree with each of the following statements?

(1 = *completely disagree*; 7 = *completely agree)*

**High standards**

1. Before I make a choice, I try to consider as many options as possible.
2. No matter what I do, I have the highest standards.
3. I never settle for second best.

**Alternative search**

1. Although I am satisfied with my current job, I try to look for better opportunities.
2. When I listen to the radio, I tend to frequently change channels.
3. When I watch TV, I channel surf, watching many programs at the same time.
4. I think the success of marriage is proportional to the number of relationships.
5. I’m a big fan of ranking things.
6. I have dreamed about living in ways that are quite different from my current life.

**Decision difficulty**

1. Choosing a gift for a friend feels like a task.
2. It is hard to choose which movie to watch.
3. When shopping, I look around as many options as possible.
4. I have a lot of trouble finding the right word when I send a text message.

## **Coping strategies**

To what extent do you agree with each of the following statements in relation to COVID-19?

(1 = *strongly disagree*; 5 = *strongly agree)*

**Preventive measures**

1. Hand hygiene

*I’ve been practicing hand hygiene (e.g., washing hand, use hand sanitizer) strictly to prevent infection.*

1. Mask-wearing

*I’ve been wearing a mask to prevent from the COVID-19.*

1. Social distancing

*I’ve been actively practicing social distancing to prevent infection.*

**Cognitive appraisal**

1. Psychological distancing

*I’ve been comforting myself by thinking “this is not a big deal”.*

1. Positive reappraisal

*I’ve been trying to view the COVID-19 outbreak in a positive way.*

1. Acceptance

*I’ve been trying to accept the reality of the COVID-19 outbreak as it is.*

**Social connection**

1. *I’ve been communicating with my family and friends via phone/texts more than usual.*

**Self-distraction**

1. *I’ve been doing other activities to take my mind off COVID-19.*

* All materials are written in Korean and the materials are translated for the purpose of presentation.
